# Suprasubduction geochemical dataset of ultramafic minerals in Southern Iran: The Ab-Bid complex

**DOI:** 10.1016/j.dib.2025.111919

**Published:** 2025-07-25

**Authors:** Mahdieh Mohammadi, Hamid Ahmadipour, Abbas Moradian, Daniele Brunelli, Reza Derakhshani

**Affiliations:** a Department of Geology, Shahid Bahonar University of Kerman, Kerman 7616913439, Iran; b Dipartimento di Scienze Chimiche e Geologiche, Università di Modena e Reggio Emilia, Italy; c Istituto di Scienze Marine ISMAR-CNR 40129 Bologna, Italy; d Department of Earth Sciences, Utrecht University 3584CB Utrecht,

**Keywords:** Mantle Wedge, Mineral Chemistry, LA-ICP-MS, Electron Microprobe, Trace Elements, Petrogenesis, Open-System Melting, Melt–Rock Interaction

## Abstract

This article presents a curated dataset of major and trace element compositions from ultramafic minerals in the Ab-Bid complex, an ophiolitic massif within the Esfandagheh–Hadji Abad mélange zone in Southern Iran. Samples were collected from orthopyroxenite dykes and their host peridotites. Major element analyses were performed using wavelength-dispersive electron microprobe analysis (EPMA), and trace elements were measured via laser ablation inductively coupled plasma mass spectrometry (LA-ICP-MS). The dataset comprises six structured Excel tables covering orthopyroxene, clinopyroxene, olivine, and spinel compositions, including rare earth and high field strength element distributions. Analytical metadata such as spot identifiers, standardization protocols, and operating conditions are included to ensure reproducibility. The data facilitate applications in melt–rock interaction modeling, mineral thermometry, and suprasubduction zone geochemical comparison. Researchers interested in mantle processes, subduction-related metasomatism, or petrological database development will find this dataset particularly valuable.

Specifications Table

**Table:** 

Subject	Earth & Environmental Sciences
Specific subject area	Suprasubduction mantle geochemistry; ultramafic petrology; mineral trace element analysis
Type of data	Table; Raw; Analyzed; Electron microprobe data; LA-ICP-MS trace element data; Spreadsheet (Excel .xlsx).
Data collection	Major elements were analyzed using a Cameca SX50 electron microprobe (EPMA) at the University of British Columbia. Trace elements were measured by LA-ICP-MS (New Wave UP213 laser coupled with Thermo Fisher X Series 2 ICP-MS) at the University of Modena. Standards (NIST SRM 610/612/614) were used for calibration. Spot sizes ranged from 5 to 80 µm. Sample selection focused on well-preserved orthopyroxenite and peridotite minerals from the Ab-Bid ultramafic complex.
Data source location	Ab-Bid ultramafic complex, Esfandagheh–Hadji Abad ophiolite mélange, Southern Iran. Sampling was conducted across an area bounded approximately by 28°4′20″ to 28°9′23″ N latitude and 56°47′29″ to 56°57′15″ E longitude. All physical samples and polished thin sections are stored at the Department of Geology, Shahid Bahonar University of Kerman, Kerman, Iran.
Data accessibility	Repository name: Mendeley Data [1] Data identification number: 10.17632/mztwzbd3yh.1 Direct URL to data: https://data.mendeley.com/datasets/mztwzbd3yh/1 Instructions for accessing these data: No restrictions. The dataset includes six structured Excel (.xlsx) sheets with: Major element compositions in wt %, Trace element in ppm.
Related research article	M. Mohammadi, H. Ahmadipour, A. Moradian, D. Brunelli, R. Derakhshani, Multistage petrogenesis and suprasubduction metasomatism of orthopyroxenites in the Ab-Bid ultramafic complex (Iran): Insights from open-system mantle melting, Lithos (2025) 108,163. https://doi.org/10.1016/J.LITHOS.2025.108163. [2].

## 1 Value of the Data

• These data provide comprehensive major and trace element compositions for key ultramafic minerals — orthopyroxene (MgSiO₃), clinopyroxene (CaMgSi₂O₆), olivine ((Mg,Fe)₂SiO₄), and spinel ((Mg,Fe)(Cr,Al)₂O₄) — collected from a suprasubduction ophiolitic complex in southern Iran. They offer a rare mineral-scale view into the geochemical architecture of a forearc mantle setting, with relevance for petrologists, geochemists, and tectonic researchers.
• Researchers can reuse these data for global comparisons across subduction-related and abyssal ultramafic suites. The dataset allows recalculation of mineral geothermometers, trace element partitioning, and modeling of melt–rock interaction processes in mantle lithologies.
• The dataset is suitable for integration into mineralogical and geochemical databases (e.g., PetDB, GEOROC), and it enhances the coverage of underrepresented tectonic regions such as the Tethyan ophiolite belt.
• Graduate students, instructors, and early-career researchers may benefit from this dataset for teaching purposes or as input for geochemical modeling tools such as MELTS, REEBOX PRO, pMELTS, PHREEQC, AQUACHEM, etc.
• All data were acquired using standardized protocols (EPMA and LA-ICP-MS) and are traceable to international reference materials, ensuring reproducibility, comparability, and long-term scientific value.

## 2 Background

The dataset was compiled to characterize the mineral-scale geochemical signatures of ultramafic rocks from the Ab-Bid complex, located within the Esfandagheh–Hadji Abad ophiolitic mélange in southern Iran. The complex is part of the Tethyan ophiolite belt, which represents ancient subduction-related lithosphere [3,4]. Ultramafic intrusions such as orthopyroxenites, particularly those with suprasubduction affinity, remain underrepresented in global geochemical compilations [5]. This dataset was generated to document the major and trace element chemistry of key mantle minerals—orthopyroxene, clinopyroxene, olivine, and spinel—using high-resolution methods (electron microprobe and LA-ICP-MS) [6,7]. Data collection focused on capturing core-to-rim variability, mineral associations, and elemental ratios relevant to melt extraction and metasomatic processes.

This data article supports the accompanying research article published in LITHOS [2], which explores melt–rock interaction and open-system melting scenarios in the Ab-Bid complex. By making the raw and processed mineral data publicly available, the data article provides transparency, facilitates reanalysis, and allows future comparisons with other ultramafic complexes in similar tectonic settings.

## 3 Data Description

The dataset is provided as a single Excel file titled: Data.xlsx

This file contains six worksheets, each corresponding to a specific mineral phase or analytical technique. The sheets include both raw and processed geochemical data obtained by EPMA and LA-ICP-MS methods. Below is a detailed breakdown of each worksheet:

Sheet 1 — Orthopyroxene

• Contains major element oxide concentrations (wt %) of orthopyroxene crystals.
• Columns represent different analytical points labeled by sample code and spot position (e.g., “E27 Core”, “E27 Rim”).
• Rows include elements such as SiO₂, Al₂O₃, Cr₂O₃, FeO, MgO, CaO, and MnO.
  Sheet 2 — Clinopyroxene
• Contains EPMA major element data for clinopyroxene from different lithologies.
• Columns are labeled by sample code and analysis type (e.g., “C14 Rim”).
• Includes the same oxide variables as in Sheet 1.
  Sheet 3 — Olivine
• Includes major element data for olivine grains from various samples.
• Columns correspond to analytical spots labeled by sample ID.
• Variables include SiO₂, MgO, FeO, MnO, and NiO.
  Sheet 4 — Spinel
• Presents EPMA-derived compositions of chrome spinels.
• Column headers identify grain IDs (e.g., “E27-cr6”, “E27-cr9”).
• Major variables include TiO₂, Cr₂O₃, Al₂O₃, MgO, and FeO.
  Sheet 5 — Clinopyroxene trace elements
• Contains trace element data (ppm) for clinopyroxene from multiple samples.
• Elements include rare earth elements (REEs) such as La, Ce, Nd, Dy, and Lu, as well as HFSE and LILE (Zr, Sr, Ti, etc.).
• Data obtained via LA-ICP-MS. Rows are organized by sample and mineral phase.
  Sheet 6 — Orthopyroxene trace elements
• Includes LA-ICP-MS trace element data for orthopyroxenes.
• Similar format to Sheet 5.
• Provides concentrations of REEs and selected incompatible elements (e.g., Zr, Nd, Sr, Ti).

All sheets use consistent column formatting: the first few rows include sample IDs, mineral phase names, and spot labels, followed by geochemical values. Missing values are represented as blank cells. Analytical metadata (spot names, rim/core identifiers) are embedded in column headers for traceability.

The entire dataset is available in .xlsx format, ensuring compatibility with most data processing software (e.g., Excel, R, Python, MATLAB). File organization supports quick extraction of data subsets by mineral phase, sample code, or analytical method.

Visual summaries (e.g., elemental range tables or radar plots) can be created from this structured file, although such representations are not included in the repository.

## 4 Experimental Design, Materials and Methods

### 4.1 Sample collection and preparation

**Fig. 1:**
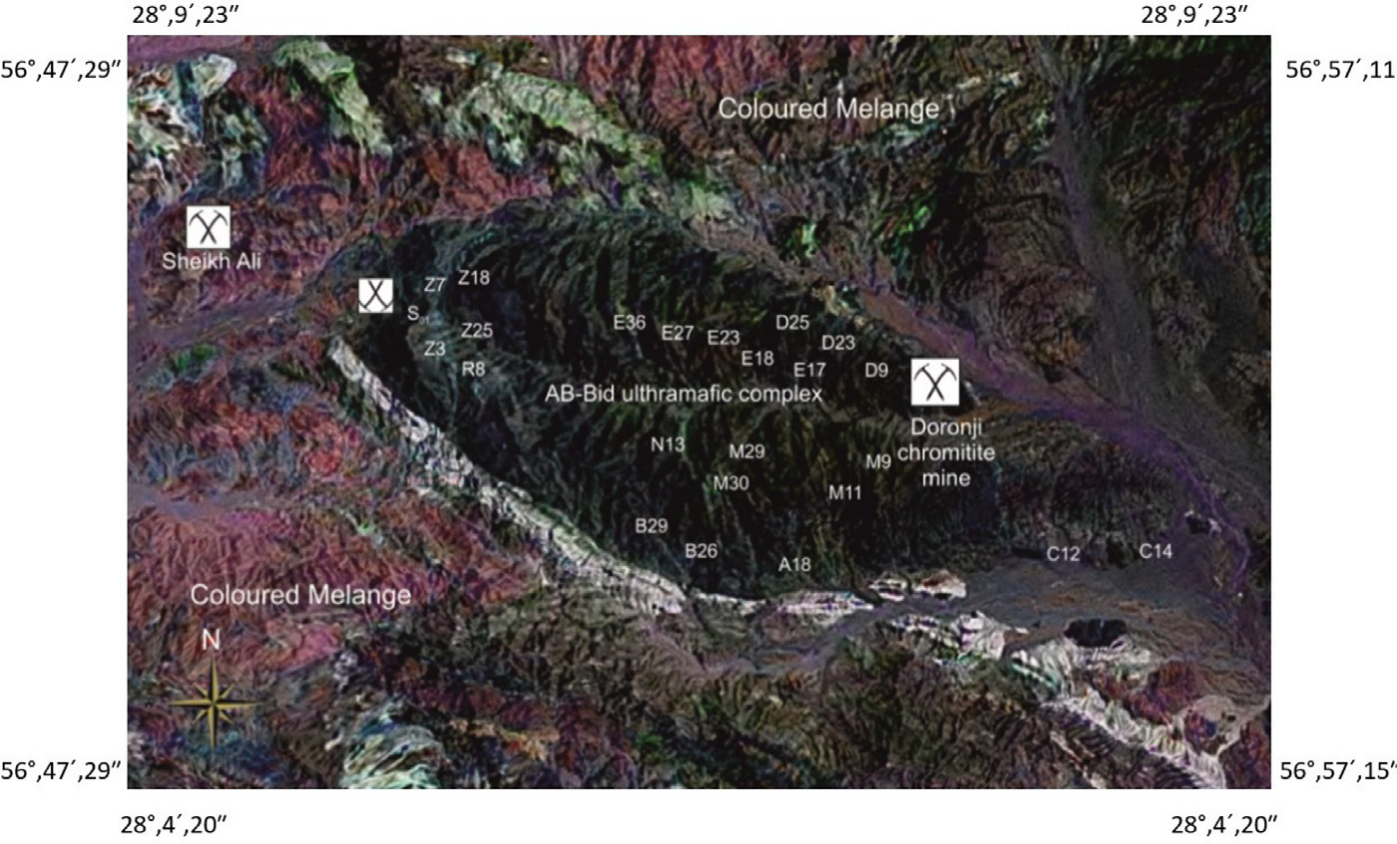
GIS-based satellite map of the Ab-Bid ultramafic complex in the Esfandagheh–Hadji Abad ophiolitic mélange, Southern Iran. Sample locations (labeled by sample codes) are shown across the massif. Coordinates were recorded in the field using handheld GPS and plotted using ArcGIS. Major geological units and nearby chromitite mines (Sheikh Ali and Doronji) are also indicated.

Rock samples were collected from the Ab-Bid ultramafic complex in the Esfandagheh–Hadji Abad ophiolitic mélange, southern Iran (Fig. 1), during a single field campaign in July–August, which corresponds to the dry summer season in the region. This timing was intentionally selected to ensure optimal exposure of outcrops and minimize the influence of surface weathering or runoff-related alterations. As seasonal effects (e.g., moisture or vegetation cover) are negligible during this period, no significant seasonal variation was expected to influence sample quality.

Sampling focused on well-preserved orthopyroxenite dykes and their host harzburgites and lherzolites. Fresh surfaces were exposed using a geological hammer, and samples were georeferenced and labeled on-site. Back in the lab, samples were cut into polished thin sections, and mineral separates were selected for in-situ analyses based on petrographic criteria. No mechanical alteration or polishing residues were observed under optical microscopy.

After petrographic examination, selected mineral grains were either analyzed in polished thin sections or extracted as individual mineral separates mounted in epoxy resin. For resin-mounted samples, grains were embedded in epoxy, ground, and polished to expose flat surfaces suitable for microanalysis. All analyses were conducted on planar, polished surfaces, with no exposed free surfaces or unprepared grain faces. Both sample types were carefully examined under reflected light microscopy to ensure the absence of contamination, inclusions, or polishing artifacts prior to EPMA and LA-ICP-MS measurements.

### 4.2 Major element analysis — EPMA

Major element concentrations in orthopyroxene, clinopyroxene, olivine, and spinel were determined by wavelength-dispersive electron microprobe analysis (EPMA) at the University of British Columbia (Canada) using a Cameca SX50 instrument equipped with four wavelength-dispersive spectrometers (WDS) and a SAMx EDS system.

• ***Operating Conditions:***
  ○ *Acceleration voltage: 15* kV
  ○ *Beam current: 20 nA*
  ○ *Spot size: 5* µm
  ○ *Peak count time: 20* s
  ○ *Background count time: 10* s
• ***Standards and Calibration:***
  ○ *International and synthetic oxide standards used:*
    ■ *SiO₂ (diopside), Al₂O₃ (kyanite), MgO (olivine), CaO (diopside), Cr₂O₃ (synthetic), FeO (fayalite), MnO (rhodonite), TiO₂ (rutile), NiO (synthetic), Na₂O (jadeite), K₂O (orthoclase).*
  ○ *Matrix corrections were applied using the ZAF method.*

Multiple point analyses were performed on each grain, including both core and rim regions, with data averaging done post-measurement.

### 4.3 Trace element analysis — LA-ICP-MS

Trace element concentrations in orthopyroxene and clinopyroxene were measured using Laser Ablation Inductively Coupled Plasma Mass Spectrometry (LA-ICP-MS) at the Centro Interdipartimentale Grandi Strumenti (CIGS), University of Modena and Reggio Emilia (Italy).

• **Instruments Used:**
  ○ Laser system: New Wave UP213 (Nd:YAG, 213 nm wavelength)
  ○ ICP-MS: Thermo Fisher Scientific X Series 2 (quadrupole)
• **Operating Conditions:**
  ○ Spot diameter: 80 µm (pre-ablation at 100 µm)
  ○ Laser fluence: 25 J/cm²
  ○ Repetition rate: 20 Hz
  ○ Dwell time: 90 s (30 s background, 60 s ablation)
• **Internal Standard and Calibration:**
  ○ Internal standard: ²⁹Si (from EPMA data)
  ○ Calibration: NIST SRM 610, 612, and 614 glasses
  ○ Data reduction: PlasmaLab software (Thermo)
  ○ Precision: 2–6 % (1σ); Accuracy: <15 % for most elements

*Trace element measurements included rare earth elements (La–Lu), high field strength elements (Zr, Ti, Hf), and large-ion lithophile elements (Sr, Ba).*

### 4.4 Data handling and processing

• All raw data were exported in spreadsheet format from instrument-specific software.
• Trace element values were normalized to chondritic or primitive mantle values using published standards (e.g., [8]).
• No post-acquisition filtering or smoothing was applied to the raw element data.
• Each Excel worksheet includes the original oxide or trace element values in weight percent or ppm, organized by mineral phase and analytical spot.

## Limitations

While this dataset provides detailed major and trace element compositions for ultramafic minerals from the Ab-Bid complex, several limitations should be noted. The number of analyzed grains per mineral phase is constrained by the availability of well-preserved crystal domains suitable for both EPMA and LA-ICP-MS. Some mineral phases, particularly olivine and spinel, are represented by fewer analyses due to alteration or grain size limitations. In a few cases, low signal intensities during LA-ICP-MS analyses resulted in below detection limit values for selected trace elements, particularly in heavy rare earth elements (HREEs) in orthopyroxene. Although analytical conditions were standardized and calibration procedures carefully followed, slight matrix effects or beam overlap in zoned grains may introduce minor variability. The dataset focuses on representative samples and does not aim for full spatial coverage across the entire complex.

## Ethics Statement

The authors confirm that they have read and follow the ethical requirements for publication in Data in Brief. The current work does not involve human subjects, animal experiments, or any data collected from social media platforms.

## CRediT authorship contribution statement

**Mahdieh Mohammadi:** Data curation, Investigation, Methodology, Formal analysis, Writing – original draft. **Hamid Ahmadipour:** Supervision, Resources, Project administration, Writing – review & editing. **Abbas Moradian:** Supervision, Resources, Validation, Writing – review & editing. **Daniele Brunelli:** Methodology, Validation, Visualization, Writing – review & editing. **Reza Derakhshani:** Conceptualization, Funding acquisition, Visualization, Writing – review & editing.

## Data Availability

Mendeley DataMajor and Trace Element Dataset of Ultramafic Minerals from the Ab-Bid Complex (Iran): Orthopyroxenite and Host Peridotite Compositions (Original data).
